# Application of Citizen Science to Sheep as a Model to Sensitize Young Citizens to Biodiversity, Animal Welfare and the Social Utility of Research

**DOI:** 10.3390/ani15020201

**Published:** 2025-01-14

**Authors:** Sara Moscatelli, Anna Paniccià, Elisa Palmioli, Laura Del Gobbo, Francesca Mercati, Paola Scocco

**Affiliations:** 1 International School of Advanced Studies, PhD Course “One Health” University of Camerino, Via Madonna delle Carceri 9, 62032 Camerino, Italy; sara.moscatelli@unicam.it; 2 School of Biosciences and Veterinary Medicine, University of Camerino, Via Pontoni 5, 62032 Camerino, Italy; anna.paniccia@studenti.unicam.it (A.P.); lauradelgobbo1995@gmail.com (L.D.G.); 3 Department of Philosophy, Social Sciences, and Education, PhD Course “Ethics of Communication, Scientific Research and Technological Innovation” University of Perugia, Piazza G. Ermini, 1, 06123 Perugia, Italy; elisa.palmioli@dottorandi.unipg.it; 4 Department of Veterinary Medicine, University of Perugia, Via San Costanzo 4, 06126 Perugia, Italy; francesca.mercati@unipg.it

**Keywords:** biodiversity, sheep, anatomical parameters, adipokines, sustainability, citizen science, attitude questionnaire, maximum performance test

## Abstract

Citizen science activities were performed using sheep as an animal model and involving 252 students aged between 9 and 11 years. The study focused on three pillars: hill/mountain landscape biodiversity, animal welfare and the social utility of research. Two types of tests—“attitude questionnaires” (AQs) and “maximum performance tests” (MPTs)—were administered. The AQ was administered before and at the end of the project to compare the children’s sensitivity to the treated topics and to assess their awareness of the project’s pillars. For each AQ item children expressed their agreement/disagreement by choosing from five response modes. The MPT aim was to evaluate if children had understood the explained topics by true/false options in respect to a sentence. The correlations between MPT scores and school evaluations for science subjects were analyzed. Among the fifteen AQ items specific to the pillars, four items related to biodiversity, three items related to animal welfare and two items related to the social utility of research, showing a significant difference (*p* < 0.05) for the second AQ’s responses. Two items showed sex-related differences. The analysis of the data from the MPT showed that the didactic methodology used was effective and improved the position of most children by bringing them into higher science evaluation groups.

## Simple Summary

This study, based on citizen science activities, aims to explore children’s knowledge and level of awareness of three concepts: biodiversity in hill and mountain landscapes, animal welfare and the social utility of research; it was performed through a series of educational activities followed by the administration of “maximum performance tests” to assess the effectiveness of the method used. The activities were structured to provide children with a progressive understanding of topics ranging from the conservation of biodiversity through sheep grazing, to the recognition of sheep breeds through anatomical differences, and culminating in the knowledge of anatomical parameters and marker molecules that can be used to assess animal welfare status. To evaluate the children’s sensitivity about the treated topics, an “attitude questionnaire” was administered prior to the beginning and at the end of the project. Tests response analysis showed both the children’s awareness of the topics increasing and the effectiveness of dissemination in citizen science activities. The children expressed their thoughts and appreciation through pictures and nursery rhymes, and someone expressed their intention to care for animals, protect biodiversity and become a scientist.

## 1. Introduction

The term “citizen science” was coined and defined by Rick Bonney in the United States and Alan Irwin in the United Kingdom [1]. However, it is still difficult to formulate an unambiguous definition of the term. According to Bonney [2], citizen science is the successful combination of public engagement with professional science projects that include both outreach and research objectives. He sees the movement as science projects in which non-scientists—the amateurs—provide observational data to scientists—the experts in the field—and ultimately gain scientific expertise through their participation.

Wiggins and Crowston [3] classified citizen science activities into five different categories based on their specific purposes: Action, Conservation, Investigation, Virtual and Education. The first typology includes all those projects of local interest with a bottom-up approach in which scientists are consultants; Conservation citizen science is closely linked to the management and conservation of natural resources. The third typology encompasses projects that require the collection of data from the physical environment, with the aim of providing scientifically valid data; Virtual citizen science is generally based on the use of virtual platforms for analysis or data collection by participants; finally, the Education category is the only one among the five that has awareness raising and involvement as its main objectives [4].

In recent years, the use of science dissemination activities aimed at children and focusing on environmental science has increased [5]. The reason for the preference for nature-related topics can be traced back to the fact that knowledge of the environment and connection to nature are fundamental to developing an environmentally aware future citizen [6,7,8,9]. In particular, many activities focus on explaining and exploring the concept of biodiversity [10,11,12].

The term “biodiversity” is a contraction of “biological diversity”, and it was used for the first time by the American biologist Walter G. Rosen during the “National Forum on Biodiversity” in 1986 [13]. Although this word is widely used, it does not have a clear and univocal definition [14]. For this reason, three main categories or types of biodiversity are generally considered: genetic diversity, referred to as the diversity of genes within a single species as well as between species; species diversity, which is the most commonly used and is based on the different taxa contained within an ecosystem; and last, functional diversity, which involves the different roles played by organisms in the ecosystem [14].

From a scientific dissemination point of view, biodiversity can be defined as the richness of animal and plant species that live in an ecosystem. An ecosystem is a part of a territory where typical animals and plants live in harmony and balance, finding the conditions for nutrition, protection and reproduction.

In the National Park of the Sibillini Mountains, there is a floristic richness of about 2200 plant species [15]. Seventy percent of these plant species are found in grassland ecosystems and 30% of them are rare or endemic; thus, grassland ecosystems represent important biodiversity hotspots [16,17,18]. Scientific studies dealing with the evaluation and comparison of the amount of plant species on ungrazed and actively grazed pastures report a higher number of plants on actively grazed pastures; in addition, better vegetative community structures were observed. Thus, grazing activity is necessary to maintain good quality grassland [19]. Therefore, the intensity of grazing required, dependent on the density of livestock, means that appropriate management is required to maintain the floristic–vegetative diversity of pasture. This can be achieved through appropriate zootechnical utilization of grassland as a trophic resource for livestock, based on the philosophy of conservation management [20], which leads to the preservation of biodiversity and respect for animal welfare while pursuing the goal of environmental sustainability. However, increasing summer aridity due to climate change has a negative impact on the nutritional quality of pastures, which is no longer adequate to support the production capacities of animals. As a result, the quality and quantity of derived products (milk, cheese, wool and meat) are decreasing [21,22].

This scenario has a negative impact on farmers’ incomes, which are insufficient to cover the costs of livestock farming. Farmers could then be forced to give up grazing, triggering an inevitable process of biodiversity loss. So, it can be possible to have good environmental sustainability if it is possible to also ensure good economic sustainability for farmers and, in turn, territories’ social sustainability. Only when there is a balance among these three aspects can we assert that a self-virtuous circle dealing with sustainability exists [22,23].

In this scenario, the application of a multidisciplinary approach to the implementation of projects that address the above issues could be a possible solution [24]. Scientific dissemination of these issues in synergy with actions that lead to the valorization of animal-derived products is necessary for adequate information and training of all the actors involved in the system, so that the actions of all these actors can lead to the implementation of ecosystem services.

Ecosystem services are the types of functions and processes performed by ecosystems that generate multiple benefits derived directly or indirectly from them and are essential for human survival and well-being [25,26]. Referring to the pastoral economy of the Sibillini area, the actors involved are farmers and consumers in addition to trade associations, students who are being trained as professionals in these fields (such as veterinarians, animal producers, and naturalists), teachers themselves, and so on [27,28,29]. The longer people have been educated about environmental and sustainability issues, the greater their awareness and the more natural and appropriate their behavior [30]; so, to educate and sensitize children to the sustainability aspects could provide good hope for the future [6,31,32].

This observational study can be considered as an example of a citizen science activity focused on nature conservation and science education [3], since it has awareness-raising and involvement as its main objectives. The main aim is to explore children’s knowledge and level of responsiveness to some environmental issues, such as the concept of biodiversity, the importance of animal welfare and how this can affect animal production capacity, and, finally, the social utility of research. In addition, the study attempts to evaluate the effectiveness of the method used on children’s learning. The long-term goal of the project is to raise children’s awareness of the environment in order for them to be conscious future consumers of animal products.

## 2. Materials and Methods

### 2.1. Sampling and Approval

The observational study was carried out in nine primary schools pertaining to three comprehensive institutes located in the Province of Macerata (Italy), chosen on the basis of their similar numbers of students living in three analyzed environments (urban areas, suburban areas and rural areas). The observational study involved 252 students of both genders, specifically children aged between 9 and 11, comprising 138 males and 114 females chosen on the basis that the children, despite their young age, were capable of understanding the contents because they already had basic knowledge of the specific topics of the pillars, having covered them in the school subject science [33,34,35]. Of the 252 children, 94 came from urban areas, 79 from suburban areas and 79 from rural areas.

In accordance with ethical guidelines, the study complies with all ethical requirements for research involving children. The project was in fact approved by the Bioethics Committee of the University of Perugia before activities in the schools began. Moreover, the “Informed Consent Form”, a document explaining the observational study in detail, was provided to parents to ensure their permission for their children to participate in completing the questionnaires. During the questionnaire process, students’ privacy was respected and safeguarded by assigning each student an alphanumeric identification code, omitting their name.

### 2.2. Activities Organization

**Figure 1:**
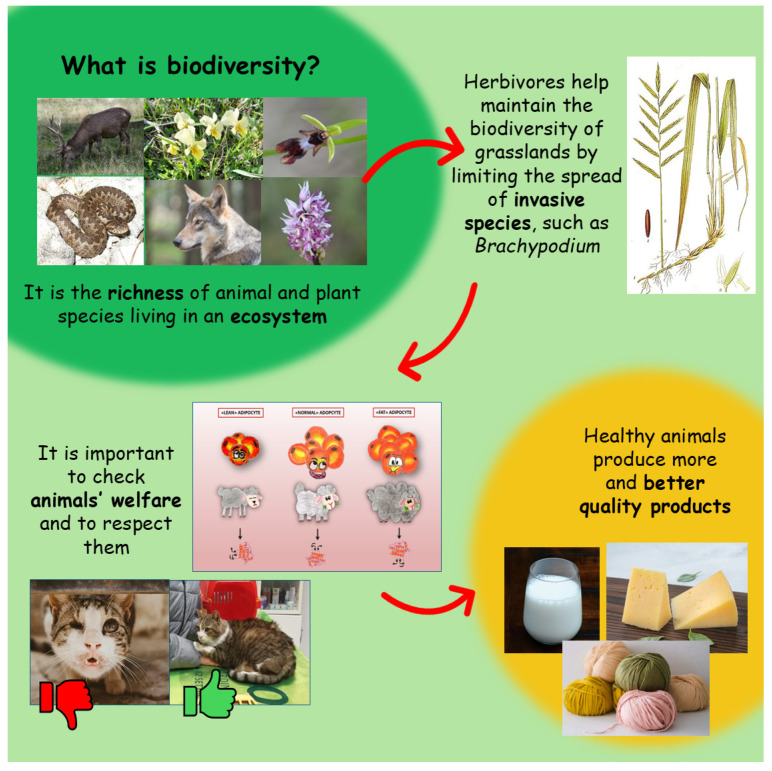
An example of PowerPoint presentation contents.

**Figure 2:**
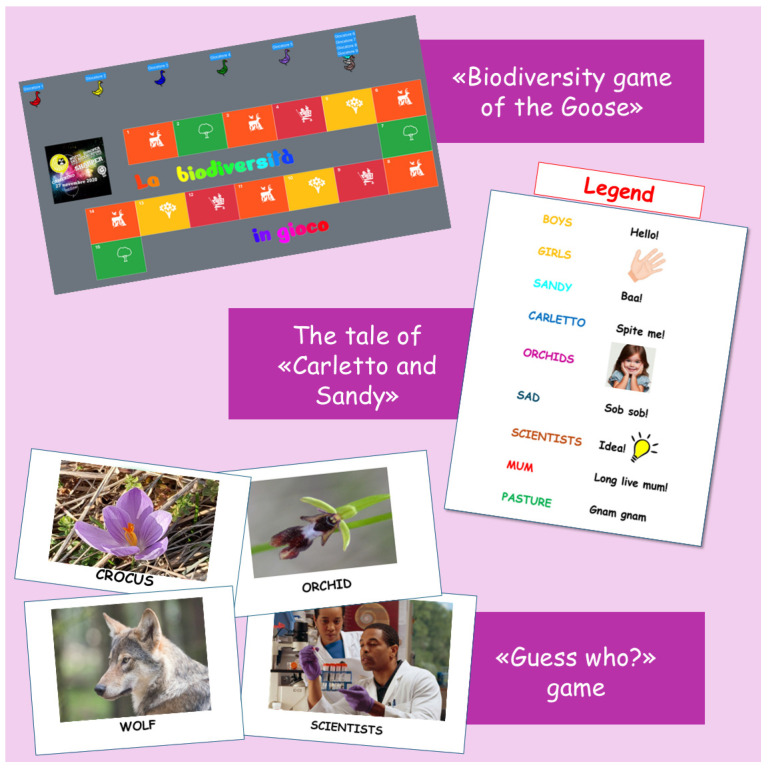
Scheme illustrating the games proposed.

The observational study was organized through a series of educational activities combined with the administration of tests to children. Educational activities were carried out during three meetings (2 h each) using interactive PowerPoint presentations about the project topics (Figure 1), followed by a ludic activity (Figure 2) to enable the children to review the concepts discussed during the theoretical part and to improve their knowledge about them [36]. All the meeting activities were carried out by the researchers.

In each meeting a main topic was covered, explaining different aspects and a playful activity linked to it was performed as follows:

First meeting: “The biodiversity of the hilly and mountain landscape”. The concepts of biodiversity and different ecosystems (agricultural, forest and grassland) were introduced. For each ecosystem, the key plant and animal species were described, highlighting their differences. The importance of certain flowers as indicators of ecosystem health was emphasized, noting that rare or protected species should not be collected. Animal groups were discussed based on characteristics such as being diurnal or nocturnal, their feeding habits (omnivores, carnivores and herbivores), and their roles as prey, predators or whether they were native or non-native species. The “Biodiversity game of the Goose” was proposed, a trivia-based game where each box hides plant species, animals, or products discussed during the lesson just completed. Children answered questions related to each box to move forward and each box also included extra trivia and curiosities not previously covered to keep the game engaging and fun.

Second meeting: “The role of animals in maintaining biodiversity and respecting animal welfare”. The use of grassland ecosystems for livestock farming was discussed, focusing on the role of sheep in maintaining biodiversity and the importance of respecting animal welfare. Children learned that an animal’s well-being depends not only on proper feeding but also on ensuring their five freedoms [37], which must be upheld by the farmer or owner. In the second meeting the “Interactive reading of the tale of Carletto and Sandy” was performed. The children participated in the storytelling, which included specific keywords that prompted them to act with sounds and movements. The interactive reading required both children and researchers to impersonate some of the human figures, animals and plant species introduced during the extension activities. The tale derived from a research project previously carried out, so the story was enriched by many scientific concepts [38].

Third meeting: “Animal welfare, product quality and the farmer’s income”. Different sheep breeds were described, focusing on their physical characteristics and suitability for different types of production (meat, wool and milk). The link between animal welfare, the quality and quantity of derived products, and the farmer’s income were emphasized. Adipokines were introduced as molecules related to animal welfare, explaining both how they change with body status and their role in regulating organ functions related to productivity [39]. Complex concepts were clarified using images and short videos that illustrated the function of organs or systems based on adipokine levels. The “Guess who?” game was used in the last meeting. Children had to ask targeted and precise questions to the researcher to obtain as much useful information as possible to guess a “mystery character”. The questions had only binary answers: yes/no. The children were divided into teams and each correct answer earned points, with the team accumulating the most points winning the game. The competitive atmosphere encouraged greater participation from the children.

During the period of activities, the teachers worked with the children to produce different types of deliverables, such as drawings and nursery rhymes, etc.

### 2.3. Tests

To assess both the effectiveness of the method used and the children’s knowledge and responsiveness to the treated topics, two different types of tests referred to as “attitude questionnaire” [40] and “maximum performance test” were administered during the project. Children answered the tests on paper; tests were administered by the researchers and the children answered them completely independently.

The attitude questionnaire (AQ) was administered before the start and at the end of the project to understand children’s sensitivity to the treated topics and to assess their awareness of the project’s pillars. In addition, it was used to evaluate differences between their responses before and after participating in the project.

The questionnaire consisted of 15 items, for each of which the children were asked to indicate their level of agreement/disagreement according to a 5-point Likert scale (totally disagree, somewhat disagree, uncertain, fairly agree, totally agree). Each response mode was given a score from 1 to 5 to obtain a numeric score to be used in the statistical analysis. To avoid misunderstandings when completing the AQ, teachers previously had the children take a test on other topics. To avoid creating a positivity bias [40], the items were formulated in two modalities:

- STRAIGHT: a positive attitude is expressed by agreement with the item. Example: “I enjoy playing outside”.
- REVERSE: a positive attitude is expressed by disagreement with the item. Example: “I do not enjoy playing outside”.

Items were divided into three groups according to the three pillars, as reported in Table 1.

The maximum performance test (MPT) aimed to evaluate whether children understood the topics covered during the meetings in the short term and was administered at the end of each meeting after the proposed game [41,42]. This type of test was chosen because the children had a certain familiarity with it acquired during activities in different museums of the territory. Each test included statements related to specific aspects covered during the meeting, and participants had to choose between “true” or “false” options. A total of 20 statements were presented to the children. Statements were short and simple so as to be easily understandable to children, as in the example, “A landscape consists of many different ecosystems”.

In both tests, children were asked to indicate gender (male or female) and type of living environment (urban area, suburban area or rural area).

### 2.4. Statistical Analysis

#### 2.4.1. Analysis of Attitude Questionnaires (AQs)

The minimum total sample size (i.e., 234.7), with alpha = 0.05 and power = 0.95, was estimated using Whitehead’s method [43]. The calculation was implemented using the posamsize function of the Hmisc R-package (Version 4.7-0) [44]. Since the response variable was ordinal and in order to account for correlations within clusters of observations, Cumulative Link Mixed Models (CLMMs) were used to test for the effects of the various explanatory variables on the children’s responses. The data were analyzed in R Studio (Version 1.2.5042) using the “lme4” package [45]. A full model was constructed with Children’s ID code and school included in the random effects, while the fixed effects tested were the children’s gender, type of living environment (urban area, suburban area or rural area) and before/after the project. A reduced model was then constructed, excluding the variable “type of living environment”, since it did not significantly contribute to explaining the variability of the sample. The two models were then compared using the Akaike Information Criterion (AIC), which favored the reduced model. The category “females” of the variable gender and the category “before” of the variable “before/after” were included automatically in the intercept by R based on the alphabetical order. Responses of children who did not complete both AQs were excluded from the analysis. Significance was set at *p* < 0.05.

#### 2.4.2. Analysis of Maximum Performance Tests (MPTs)

The correlation between MPT scores and children’s evaluations of the science subject was analyzed.

According to Italian law, scholar evaluations are divided in four categories:

First acquisition
Base
Intermediate
Advanced

MPT results were divided into four categories depending on the sum of the values the children had scored on all MPT tests. Values’ ranges were:

1. 1–5
2. 6–10
3. 11–15
4. 16–20

Since the data violated the assumption for “minimum expected cell frequency”, Fisher’s exact test was used to determine if there was a significant association between the school science evaluation and the results of the MPT. Significance was set at *p* < 0.05.

The categories “first acquisition” and “1–5” were not considered in the statistical analysis because no children fell in those categories. The responses of children who did not perform one or more of the three tests were not considered during the statistical analysis.

## 3. Results

### 3.1. Attitude Questionnaire (AQ) Results

The statistical analyses performed (CLMMs) highlighted significant differences in the children’s responses after participating in the project for nine of the fifteen items, in particular for four of the five items related to biodiversity, three out of the five items related to welfare and two out of the five items related to the social utility of research. One item related to biodiversity and one related to the social utility of research showed significant differences between the genders. The results are expressed in Table 2.

#### 3.1.1. Biodiversity Items

**Figure 3:**
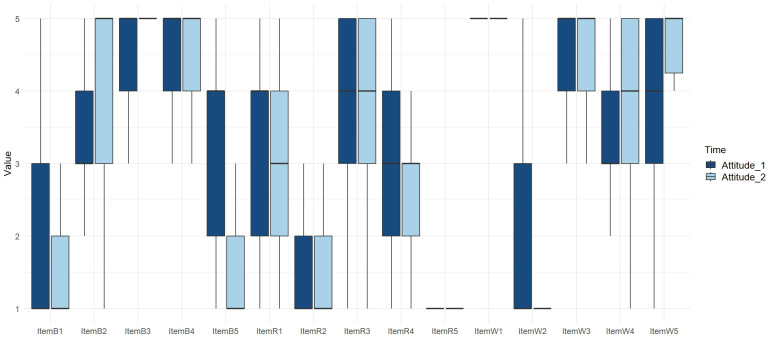
Box plots showing the distribution of scores per item, based on children’s responses in the attitude questionnaires administered before the beginning of the project (Attitude_1, dark blue) and after the end of the project (Attitude_2, pale blue). B1–B5 biodiversity items; R1–R5 social utility of research items; and W1–W5 welfare items.

**Figure 4:**
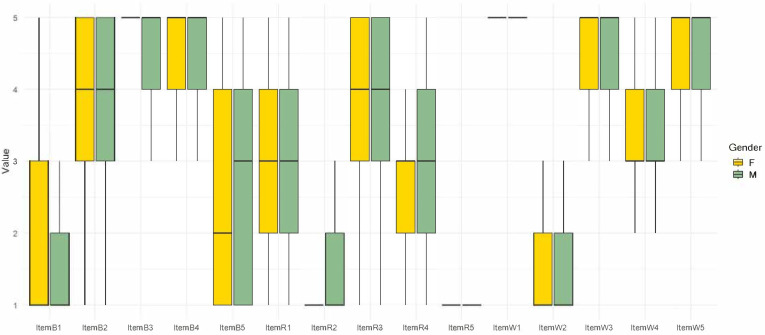
Box plots showing the distribution of scores per item for females (yellow) and males (green), based on children’s responses in the attitude questionnaires. B1–B5 biodiversity items; R1–R5 social utility of research items; and W1–W5 welfare items.

Among the five items related to biodiversity, four items showed a significant difference in the second attitude test’s responses (Figure 3), while one item showed a significant difference between the genders (Figure 4). In particular:

For the reverse item B1, the children showed a significant decrease in agreement with the idea that orchids are only found in florists’ shops (*p* < 0.001).

For straight item B2, children showed a significant increase in agreement with the statement that herbivores’ grazing promotes the presence of various flowers and grasses (*p* < 0.001).

For the straight item B3, no significant differences were observed in children’s agreement with the idea that the presence of many different animals is important for the planet (*p* = 0.2194).

For straight item B4, children showed a significant increase in agreement that a landscape contains different ecosystems (*p* = 0.0062).

For reverse item B5, children showed a significant decrease in agreement with the idea that all sheep are very similar to each other (*p* < 0.001), and a significant difference was observed between genders with males agreeing more with the item than the females (*p* = 0.0475).

#### 3.1.2. Welfare Items

Among the five items related to animal welfare, three items showed a significant difference in the second attitude test’s responses (Figure 3), while no significant differences were found between the genders (Figure 4).

For straight item W1, no significant differences were observed regarding children’s agreement that the farmer takes good care of his sheep (*p* = 0.1685).

For reverse item W2, there was a significant decrease in the children’s agreement with the statement that sheep are fine even if they do not eat much (*p* < 0.001).

For straight item W3, no significant differences were observed in agreement with the idea that a sheep has a better life if kept in open air (*p* = 0.5142).

For straight item W4, children showed a significant increase in agreement with the belief that if a sheep is sick, it does not give birth to lambs (*p* < 0.001).

For straight item W5, children showed a significant increase in agreement with the notion that if a sheep is fine, it can produce more milk (*p* < 0.001).

#### 3.1.3. Social Utility of Research Items

Among the five items related to the social utility of research, two items showed significant differences in the second attitude test’s responses (Figure 3), while one item showed a significant difference between males and females (Figure 4).

For reverse item R1, children showed a significant decrease in agreement with the belief that scientists use difficult terms when talking (*p* = 0.0348).

For reverse item R2, children showed no significant differences about the notion that they would not like to study the animals and plants in the world, but a significant difference was observed between genders (*p* < 0.001), with males agreeing more than females (*p* = 0.0537).

For straight item R3, no significant differences were observed in children’s agreement with liking the work of a scientist (*p* = 0.6691).

For reverse item R4, children showed a significant decrease in agreement with the idea that they do not want to become scientists when they grow up (*p* < 0.001).

For reverse item R5, no significant differences were observed in children’s agreement with the view that the work of a scientist is not useful (*p* = 0.9050).

### 3.2. Analysis of Maximum Performance Tests (MPTs) Results

**Figure 5:**
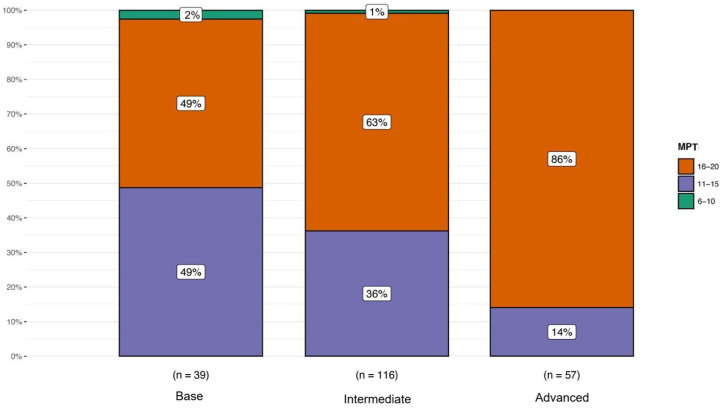
Distribution of MPT scores (6–10, 11–15, 16–20) across Base, Intermediate and Advanced levels. Numbers in parentheses indicate sample size for each group.

The exact Fisher’s test results highlighted a significant correlation between the MPT results and the evaluations of the science subject, considering the total sample (two-tailed, *p* < 0.001). As expected, among the children belonging to the Advanced group, 86% scored in the highest result range (16–20), while the remaining 14% scored in the result range 11–15. Instead, among the children belonging to the Intermediate group (expected score 11–15), 63% scored in the highest result range (16–20), while 36% of the children scored in the expected result range and the remaining 1% scored in the lowest result range (6–10). Surprisingly, among the children belonging to the Base group (expected score 6–10), 49% scored in the highest result range (16–20), 49% of the children scored in the range 11–15 and only the remaining 2% of the children scored in the expected result range (Figure 5).

**Figure 6:**
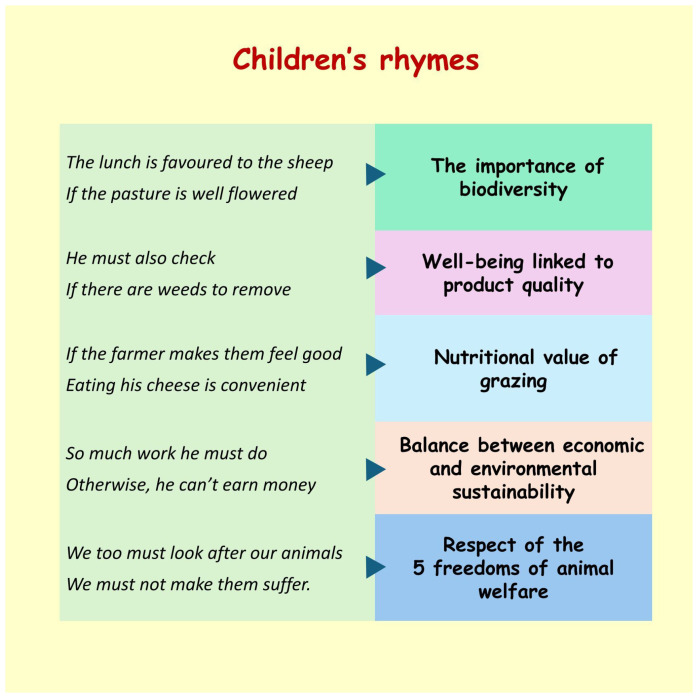
An example of nursery rhymes originally written in Italian, in which the correspondence between the sentences and the respective specific topics can be highlighted.

In addition to the objective results obtained through the administration of the tests, considerable attention was also paid to the rhymes and drawings that the children made at the end of the activity (Figure 6).

## 4. Discussion

The activities described fall under the umbrella of citizen science. The typology of science dissemination for education projects is an effective way to provide students with opportunities to engage with scientific content and research relevant to their lives and communities. The research structure follows four basic design phases [46].

Identify the science most relevant to the decisions that people face: no scientific layman can know all the scientific aspects involved in a given phenomenon. For this reason, the disseminator must identify the few scientific results that people need to know among the myriad of data available [47,48]. In our case, the audience consisted of children aged between 9 and 11 years old, who often have a certain influence on the everyday decisions of the household [49,50,51]. Since the project aimed to raise children’s awareness of biodiversity, animal welfare and the social utility of research, the likely “decision-making areas” were limited to the conscious purchase of animal products and respect and care for animals.

Determine what people already know: it is crucial not to dwell on explaining facts and notions that the audience already knows and it is fundamental to check people’s background knowledge before explaining in order to detect possible gaps in knowledge that need to be addressed [47,52].

The research team on this project had previously carried out tests in other schools that were not part of the sample universe of this study, to verify and test the effectiveness of the communication and activities that had been developed. Furthermore, before starting the cycle of meetings that the children would attend, each of them had been asked to fill in an AQ, the purpose of which was to bring out their perception of the topics that would be dealt with.

Design communications to fill the critical gaps (between what people know and need to know): once it has been established what the majority of the audience knows and what they should know, critical gaps need to be filled in [46,48,53,54]. Regarding science dissemination to children, one of the most widely adopted techniques to convey messages is the use of emotions, especially through playful activities [36]. So our lessons were carried out using colourful and image-rich PowerPoint presentations; the explanation also aimed to involve the children through the formulation of questions.

Evaluate the adequacy of those communications: the last stage in the design of scientific dissemination is verification, the proof that can confirm and approve the work that has been done or that can highlight shortcomings and aspects that have been overlooked and therefore need to be supplemented, enhanced or corrected. Poor science communication can cause serious damage because it risks eroding trust between scientists and the public, as well as devaluing the image of research and spreading wrong messages or ideas [46]. In our observational study, each meeting finished with the completion of an MPT relating to the topics covered on that day; this allowed the researchers to assess children’s instantaneous ability to take in and assimilate the notions explained. Moreover, at the end of the project, the students were given an AQ identical to the one they had completed on the first day in order to detect any changes in the children’s perceptions of the topics.

Based on the obtained results, it is possible to affirm that the AQs and MPTs were valid tools for understanding if the learning methodologies used were appropriate for scholar education, as demonstrated by the statistical significance in the comparison of the most AQ items and by the correlation of results with the MPTs.

The AQs highlighted how children increased their responsiveness and sensitivity to the different topics, with nine out of fifteen items showing significant differences in responses after the children participated in the project. All the nine items saw a decrease or increase in agreement, consistent with the straight or reverse nature of the item. This highlights how the method used was appropriate for sensitizing children to biodiversity, animal welfare and research social utility issues.

For the items that showed non-significant differences (B3, W1, W3, R2, R3 and R5), it can be hypothesized that the children were already sensitive to the topics covered. For example, for the item W3, “A sheep has a better life if kept in open air”, children agreed with the statement prior to participating in the project because they were likely to already be aware of the importance of open living conditions for animals. Similarly, for item R5, “The work of the scientist is not useful”, children disagreed with the statement before the project, as they probably believed that scientists’ work is important for society [55,56].

The children’s sensitivity about biodiversity strongly increased; they clearly understood that biodiversity depends on the richness of animal and plant species as well as on animal–plant interactions. In addition, they focused their attention on landscape biodiversity and on the importance of protecting some species that represent environmental markers.

Children clearly understood the importance of animal welfare and that it is also related to animal productive performance and to farm income.

Finally, they showed an understanding of the work of scientists; they were able to appreciate that researchers can convey scientific concepts using understandable terminology. While starting from the idea that the work of researchers is very important, they have shown a growing desire to become researchers themselves, even if becoming researchers was appreciated more than studying for the purpose.

Females and males showed similar behaviors toward the covered topics, with only two items showing significant differences between genders. For example, item R2, “I would NOT like to study the animals and plants present in the world”, had a higher level of agreement among males. However, as indicated in R4, children expressed interest in becoming scientists when they grew up. This suggests that the word “study” may have created a bias, with females displaying a greater predisposition to study compared to males [57]. We can also speculate that this assumption is further supported by the results for the B5 item “The sheep are all very similar to each other”; a slightly significant difference was evidenced, with males agreeing more to the topic then females, suggesting that females seem to have greater observation skills [58].

In the AQ, no differences were found between types of living environment, which could be related to the contexts in which children live. Children participating in the project came from small towns that are particularly close to the countryside. For this reason, in this context, it is hard to distinguish between urban, peripheral or rural areas. Probably, research developed in bigger cities, where the distinction is more defined, could reveal differences among the living contexts.

The results of the correlation between the MPT and the evaluations of the science subject highlighted that the learning method used had a positive effect. Students in the Advanced group remained quite stable; the 14% of the Advanced students that did not meet the expected score included those children who required more in-depth study to fully comprehend the concepts. Students in both the Base and Intermediate groups exceeded the expected results, with the Intermediate group showing a better performance overall. The Base group also demonstrated positive outcomes, though to a lesser degree. This improvement can be attributed to the interactive lessons that incorporated PowerPoint presentations and educational games, which facilitated greater participation among children who typically engage less in traditional classroom settings, thereby improving their performance [53,59].

It is clear that independent study following lessons provides an irreplaceable means of correctly understanding concepts [60]; however, not all children may have the same support available at this stage (e.g., books, availability of parents and availability of additional educational supports). The MPT could represent a useful tool to evaluate the immediate and unmediated understanding ability of children; in addition, it may be used as an effective teaching activity, especially for some children, and as an integrative methodology during the scholar year.

Finally, the data from the MPT showed that the didactic methodology used was effective and improved the position of most children, bringing them into higher science evaluation groups. The children expressed their thoughts and appreciation by means of drawings and nursery rhymes; in addition, some of them expressed their intention to care for animals, protect biodiversity and to become scientists.

## 5. Conclusions

The obtained results showed the effectiveness of the citizen science activities performed; this is evidenced by the children’s increased sensitivity to the proposed topics and their improved ability to immediately comprehend the concepts.

The proposed teaching method may be applied to integrating other scholar disciplines and also for different school/age segments; it is clear that the topics have to be treated by determining the contents, terminology, tests and types of games according to the specific ages of the students.

## Figures and Tables

**Table 1:** List of items divided by pillars.

Pillars		Items
Biodiversity	B1	“Orchids are only found at florists’ shops”
	B2	“The grazing activity of herbivores facilitates the presence of many types of flowers and grasses”
	B3	“The presence of many different animals is important for the planet”
	B4	“In a landscape I can see different environments”
	B5	“The sheep are all very similar to each other”
Animal Welfare	W1	“The farmer takes good care of his sheep”
	W2	“A sheep is fine if it does not eat much”
	W3	“A sheep has a better life if kept in open air”
	W4	“If a sheep is sick, it does not give birth to lambs”
	W5	“If a sheep is fine, it can produce more milk”
Research	R1	“Scientist uses difficult terms when talking”
	R2	“I would not like to study the animals and plants present in the world”
	R3	“I like the work of the scientist”
	R4	“I do not want to become a scientist when I grow up”
	R5	“The work of the scientist is not useful”

**Table 2:** CLMM results. The “female” category of the “gender” variable and the “before” category of the “before/after” variable were included automatically in the intercept by R, based on the alphabetic order.

Item	Variable	Estimate	Std_Error	z_Value	*p*_Value
B1	Gender: Males	−0.143919381	0.15973897	0.900966002	0.3676
	B/A: After	−0.537121737	0.120231146	4.467409263	**<0.001**
B2	Gender: Males	−0.157378086	0.117563738	1.338661802	0.1807
	B/A: After	0.937588617	0.109185932	8.587082563	**<0.001**
B3	Gender: Males	−0.203855542	0.179763917	−1.13401813	0.2568
	B/A: After	0.160773273	0.130912448	1.228097677	0.2194
B4	Gender: Males	0.001527483	0.133421605	0.011448542	0.9909
	B/A: After	0.300679087	0.109814837	2.738055225	**0.0062**
B5	Gender: Males	0.254132054	0.128229898	1.981847119	**0.0475**
	B/A: After	−1.533653233	0.127921007	11.98906475	**<0.001**
W1	Gender: Males	0.002804654	0.180141767	0.015569148	0.9876
	B/A: After	0.200278809	0.145441369	1.377041552	0.1685
W2	Gender: Males	−0.00819291	0.166125866	0.049317485	0.9607
	B/A: After	−0.966676784	0.141669581	6.823460443	**<0.001**
W3	Gender: Males	0.120955437	0.1347983	0.897306848	0.3696
	B/A: After	0.076199385	0.1168067	0.652354569	0.5142
W4	Gender: Males	0.120136754	0.123423603	0.973369368	0.3304
	B/A: After	0.439002023	0.101536235	4.323599568	**<0.001**
W5	Gender: Males	0.11793855	0.162287407	0.726726444	0.4674
	B/A: After	1.280417315	0.139461015	9.181184522	**<0.001**
R1	Gender: Males	0.080660971	0.14926788	0.54037728	0.5889
	B/A: After	−0.208498298	0.098778243	2.110771484	**0.0348**
R2	Gender: Males	0.606889927	0.180991804	3.353134864	**<0.001**
	B/A: After	0.238449423	0.123597739	1.929237744	0.0537
R3	Gender: Males	−0.288752749	0.175402998	1.646224712	0.0997
	B/A: After	0.043411507	0.101576964	0.427375506	0.6691
R4	Gender: Males	0.210253446	0.18196004	1.15549241	0.2479
	B/A: After	−0.314763447	0.103446074	3.042778069	**<0.001**
R5	Gender: Males	0.249414346	0.170608446	1.4619109	0.1438
	B/A: After	0.016205601	0.135745123	0.119382564	0.9050

B1–5 biodiversity items, W1–5 welfare items, R1–5 research items. A/B = after/before. Values in bold are statistically significative.

## Data Availability

The data are unavailable due to privacy and ethical restrictions.
